# Predicting pathological complete response after neoadjuvant chemotherapy in breast cancer by clinicopathological indicators and ultrasound parameters using a nomogram

**DOI:** 10.1038/s41598-024-64766-2

**Published:** 2024-07-16

**Authors:** Tingjian Zhang, Yuyao Liu, Tian Tian

**Affiliations:** 1 Department of Thyroid and Breast Surgery, The People′s Hospital of Leshan, Leshan, Sichuan Province 614000 China; 2 Department of Radiology, The People′s Hospital of Leshan, Leshan, Sichuan Province 614000 China; 3 General Surgery Department, Yongchuan Hospital of Chongqing Medical University, Yongchuan District, Chongqing, 402160 China

**Keywords:** Breast cancer, Albumin-to-alkaline phosphatase ratio, Neoadjuvant chemotherapy, Pathological complete response, Nomogram, Breast cancer, Cancer imaging, Cancer therapy

## Abstract

The study explored the impact of pretreatment serum albumin-to-alkaline phosphatase ratio (AAPR) and changes in tumor blood supply on pathological complete response (pCR) in breast cancer (BC) patients following neoadjuvant chemotherapy (NACT). Additionally, a nomogram for predicting pCR was established and validated. The study included BC patients undergoing NACT at Yongchuan Hospital of Chongqing Medical University from January 2019 to October 2023. We analyzed the correlation between pCR and clinicopathological factors, as well as tumor ultrasound features, using chi-square or Fisher's exact test. We developed and validated a nomogram predicting pCR based on regression analysis results. The study included 176 BC patients. Logistic regression analysis identified AAPR [odds ratio (OR) 2.616, 95% confidence interval (CI) 1.140–5.998, *P* = 0.023], changes in tumor blood supply after two NACT cycles (OR 2.247, 95%CI 1.071–4.716,* P* = 0.032), tumor histological grade (OR 3.843, 95%CI 1.286–10.659, *P* = 0.010), and HER2 status (OR 2.776, 95%CI 1.057–7.240, *P* = 0.038) as independent predictors of pCR after NACT. The nomogram, based on AAPR, changes in tumor blood supply after two NACT cycles, tumor histological grade, and HER2 status, demonstrated a good predictive capability.

## Introduction

BC has emerged as the most prevalent form of cancer globally, posing a significant threat to women's health and quality of life^1^. Preoperative NACT has become an essential option in the comprehensive treatment of BC patients, and its survival benefit has long been shown to be equivalent to that of postoperative adjuvant chemotherapy^2^. In addition, NACT not only achieves tumor downstaging, increases the chance of surgery, and improves the breast-conservation rate, but also provides an opportunity to assess the sensitivity of chemotherapy. This aids clinicians in promptly adjusting the treatment regimen according to the tumor response, thereby enhancing patient survival rates^3^. Numerous studies indicate that patients receiving NACT and achieving pCR tend to have a more favorable clinical prognosis^4,5^. However, BC is a heterogeneous disease, and not all patients derive benefits from NACT^6^. Delaying surgery may even increase the risk of disease progression in chemotherapy-resistant tumors. Studies examining and characterizing biomarkers for predicting pCR have been undertaken. However, most of these studies have emphasized prognostic biomarkers rather than predictors identified before treatment initiation or at NACT onset^7–9^. Consequently, there is a need to identify factors influencing pCR in BC patients after NACT and develop predictive models to identify patients most likely to benefit from NACT. Previous studies indicate that peripheral inflammation indicators, such as neutrophil-to-lymphocyte ratio (NLR), lymphocyte-to-monocyte ratio (LMR), and platelet-to-lymphocyte ratio (PLR), are linked to the prognosis of BC patients. Using these metrics, the rate of PCR in NACT patients can be predicted, though the findings are controversial^10–13^. Liver-synthesized albumin possesses immunomodulatory functions and can be considered a surrogate marker for systemic inflammation. Low albumin levels are additionally linked to an insufficient organismal anti-cancer response^14,15^. Alkaline phosphatase (ALP) is considered to be linked to systemic inflammation and tumor advancement^16,17^. The ALB-to-ALP ratio (AAPR), derived by dividing the ALB level by the ALP level, is a composite index associated with systemic inflammation and is a superior predictor of tumor prognosis compared to ALB or ALP individually^18^. AAPR is a novel marker initially identified as a prognostic factor in hepatocellular carcinoma^19^. In recent years, it has been linked to the prognosis of hepatocellular carcinoma, nasopharyngeal carcinoma, small cell lung cancer, renal cancer, and various other malignant tumors^20–24^. Although a retrospective study has shown that low AAPR values are associated with a poorer survival prognosis in BC, there is a lack of high-quality evidence to support this, and the value of AAPR values in predicting prognosis in BC is currently fraught with controversy^25^.

Despite the clinical ease of obtaining AAPR, there is a scarcity of studies utilizing AAPR to predict pCR rates in BC patients undergoing NACT. Clinical acquisition of ultrasound images of tumors is also straightforward. Patients' tumors are routinely assessed clinically before and during treatment, with breast ultrasound being the primary choice for evaluating tumors in BC patients due to its cost-effectiveness, non-invasiveness, and accuracy.Nevertheless, few studies have established predictive models for NACT efficacy based on ultrasound parameters and clinical indicators^26^. By integrating breast ultrasound with relevant clinicopathological indicators, we innovatively devised and validated a nomogram. This nomogram is based on AAPR values and changes in patients' tumor blood supply, aiming to predict the pCR in BC patients undergoing NACT.

## Methods

### Population

BC patients undergoing NACT at Yongchuan Hospital of Chongqing Medical University between January 2019 and October 2023 were included based on inclusion–exclusion criteria. Inclusion criteria were: (1) BC diagnosis confirmed by puncture pathology; (2) Completion of at least four cycles of NACT; (3) Subsequent surgery after NACT; (4) Availability of comprehensive clinicopathological and postoperative pathological data; (5) Assessment of the primary tumor using color doppler ultrasound before and after two cycles of NACT. Exclusion criteria were: (1) Antitumor therapy prior to NACT;(2) Locally advanced or distant metastases; (3) Lack of post-NACT surgery; (4) Inadequate clinicopathological and postoperative pathological data; (5) Non-assessment of the primary tumor by color doppler ultrasound before NACT or after two cycles of NACT. The study adhered to the principles of the Declaration of Helsinki and received approval from the Medical Research Ethics Committee of Yongchuan Hospital of Chongqing Medical University (No. 2023–003). The retrospective study did not involve patient privacy. Therefore, the waiver of informed consent was approved by the Ethics Committee of Yongchuan Hospital of Chongqing Medical University. The authors were not provided with information that could identify individual participants during or after data collection.

### Clinicopathologic analysis

Prior to receiving NACT, patients underwent an assessment encompassing current and past disease history, age, body mass index (BMI), menstrual status, chemotherapy cycle, tumor size, lymph node status, ALB, ALP, AAPR, NLR, LMR in peripheral blood, hormone receptor (HR) status, human epidermal growth factor receptor 2 (HER2) status, Ki-67 index, NACT regimens, and other relevant information. Receiver operating characteristic (ROC) curves were utilized to calculate the optimal cut-off values for continuous variables. Subsequently, continuous variables were transformed into binary variables using the determined cut-off values. Clinical evaluation of breast tumors, encompassing tumor size and preoperative lymph node status, relied on breast ultrasound. Estrogen receptor (ER), progesterone receptor (PgR), HER2, and Ki-67 statuses were determined through immunohistochemistry (IHC) analysis of pre-treatment core biopsy specimens. HER2 status was classified as 0, 1, 2, or 3. HER2 was considered positive with a staining intensity score of3 + and considered negative with a score of 0 or 1 + . Furthermore, FISH analysis was used to determine HER2 status in tumors with a score of 2 + . Cancers with 1–100% of cells positive for ER/PgR expression were considered ER-positive/PgR-positive. The Ki-67 index was defined as the percentage of the total number of tumor cells (at least 1000) with nuclear staining over 10 high powered fields (× 40).

PCR was defined as no residual invasive cancer in the breast post-NACT and the absence of lymph node metastases during axillary surgery (ypT0ypN0) with no evidence of disease.

### Ultrasound examination

The included patients underwent two-dimensional grey-scale ultrasound and color Doppler examinations before and after two cycles of NACT. Two breast ultrasound specialists independently reviewed all ultrasound images. Standard ultrasound characteristics of tumors in patients before and after two cycles of NACT were documented. These features encompass tumor size, morphology, borders, internal echogenicity, posterior echogenicity, Alder grading of blood flow signals, calcification, and changes in these ultrasound features.

### Statistical analyses

Statistical analyses were conducted utilizing R software (version 4.0.2) and SPSS (version 26.0). The maximum Youden index was employed to ascertain the optimal cut-off value, converting continuous variables into binary variables based on this determined threshold. Differences between the two groups were analyzed using the chi-square test or Fisher's exact test. Univariate and multivariate logistic regression analyses were employed to identify factors associated with pCR after NACT. Subsequently, based on the multivariate logistic regression analysis results, a nomogram prediction model for pCR after NACT was developed. The discriminatory performance of the nomogram was quantified through the measurement of Harrell's C-index. The model's discriminatory abilities were evaluated by measuring the area under the receiver operating characteristic (ROC) curve. A calibration curve was drawn to evaluate its calibration effect. Additionally, decision curve analysis was conducted to assess the net benefit of the nomogram. A significance level of* P* < 0.05 was considered statistically significant.

### Ethical approval and consent to participate

This study was performed in line with the principles of the Declaration of Helsinki. Approval was granted by the Ethics Committee of Yongchuan Hospital of Chongqing Medical University (No. 2023–003). The retrospective study did not involve patient privacy. Therefore, the waiver of informed consent was approved by the Ethics Committee of Yongchuan Hospital of Chongqing Medical University. The authors were not provided with information that could identify individual participants during or after data collection.

## Result

### Clinicopathological and ultrasound characteristics of BC patients received NACT

**Figure 1:**
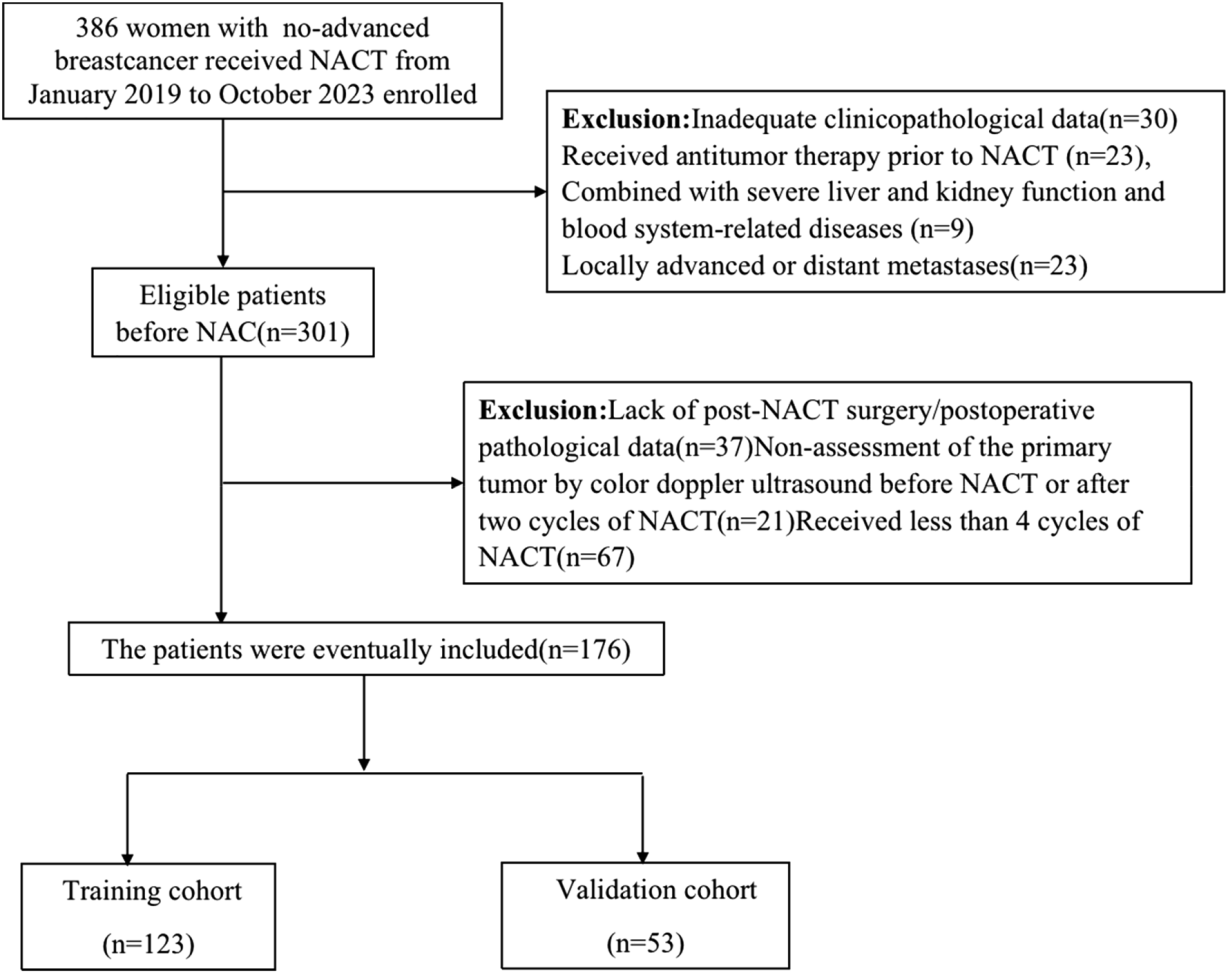
Recruitment pathway for patient selection.

Our study finally included 176 patients, with a mean age of 49.56 ± 8.65 years. (Fig. 1)The optimal cut-off value for the AAPR was 0.881. All patients underwent ultrasound assessment of the primary tumor both before and after two cycles of NACT, followed by surgical intervention post-NACT. Postoperative pathology verified that 68 patients (38.64%) achieved pCR. Tables 1, 2 present the patients' clinicopathological characteristics and the tumors' ultrasonographic features. The chi-square test showed that serum albumin concentration, histological grading of the tumor, HER2 status and changes in tumor blood supply after two therapy cycles were significantly associated with the pcr rate in bc patients after NACT.

**Table 1:** Baseline clinicopathological characteristics of patients.

Factors		Non-pCR (N = 108)	pCR (N = 68)	Total	X^2^	*P*
Clinical variable						
Age(years)	< 42.5	28	13	41	1.082	0.298
	≥ 42.5	80	55	135		
BMI(kg/m^2^)	< 24.45	63	36	99	0.493	0.483
	≥ 24.45	45	32	77		
Menopausal status	Premenopausal	52	36	88	0.383	0.536
	Postmenopausal	56	32	88		
Therapy cycle	< 6	38	18	56	1.461	0.227
	≥ 6	70	50	120		
cT	1/2	88	60	148	1.423	0.233
	3	20	8	28		
cN	N0	21	17	38	0.761	0.383
	NX	87	51	138		
ALB	< 45.25	61	28	89	3.910	**0.048**
	≥ 45.25	47	40	87		
ALP	< 73.50	67	37	104	1.004	0.316
	≥ 73.50	41	31	72		
AAPR	< 0.881	87	47	134	3.005	**0.083**
	≥ 0.881	21	21	42		
NLR	< 2.042	45	20	65	2.690	0.101
	≥ 2.042	63	48	111		
PLR	< 184.589	79	58	137	3.569	**0.059**
	≥ 184.589	29	10	39		
LMR	< 5.210	78	43	121	1.569	0.210
	≥ 5.210	30	25	55		
Pathological variable						
Histological grade	I/II	69	14	83	31.396	** < 0.001**
	III	39	54	93		
ER	Negative	38	31	69	1.895	0.169
	Positive	70	37	107		
PR	Negative	48	38	86	2.185	0.139
	Positive	60	30	90		
HER2	Negative	77	19	96	31.635	** < 0.001**
	Positive	31	49	80		
KI67	< 0.48	79	42	121	2.517	0.113
	≥ 0.48	29	26	55		
Tumor diameter reduction (cm)	< 1.05	48	28	76	0.182	0.670
	≥ 1.05	60	40	100		

BC: Breast cancer; NACT: Neoadjuvant chemotherapy; pCR: Pathological complete response; ER: Estrogen receptor; PR: Progesterone receptor; HER-2: Human epidermal growth factor receptor-2; ALB: Albumin; ALP: Alkaline phosphatase AAPR: albumin-to-alkaline phosphatase ratio NLR: Neutrophil-to-lymphocyte ratio; PLR: Platelet-to-lymphocyte ratio; LMR: Lymphocyte-to-monocyte ratio.

Significant values are in bold.

**Table 2:** Comparative analysis of differences in ultrasound between the pCR group and Non-pCR group.

Factors		Before NACT			After 2 cycles of NACT		
		Non-pCR(108)	pCR(68)	*P*	Non-pCR(108)	pCR(68)	*P*
Form	Regular	3	2	0.949	10	8	0.593
	Irregular	105	66		98	60	
Adler grade*	Level 0	8	2		9	7	
	Level 1	25	14	0.426	38	26	0.909
	Level 2	34	28		42	25	
	Level 3	41	24		19	10	
Boundary	Circumscribed	38	26	0.682	9	5	0.815
	Indistinct	70	42		99	5	
Echo	Very low	8	8		5	4	
	Low	98	58	0.631	95	58	0.914
	Mix	1	1		1	1	
	Wait for an echo	1	1		7	5	
Posterior acoustic	Shadowing	14	10	0.184	39	28	0.124
	No change	89	50		66	34	
	Enhancement	5	8		3	6	
Calcification	None	50	32	0.921	41	26	0.971
	Have	58	36		67	42	

*Adler grade: Level 0: no blood flow in the lesion; Level 1:a small amount of blood flow, with 1 or 2 punctured or thin rod blood flow; Level 2: moderate blood flow, one major blood vessel can be seen, its length is close to or beyond the radius of the lesion or 3 ~ 4 punctured or fine rod-shaped blood vessels; Level 3: abundant blood flow, visible more than 4 blood vessels or interconnected, intertwined into a network. Adler grading observes the distribution and richness of blood flow, finds the section with the most abundant blood flow, calculates the number of blood vessels. Te definition ofAlder grading change is whether grading decreases after NAC.

### Logistic regression analysis for detecting the factors related to pCR after NACT

Univariate logistic regression analyses were conducted to identify factors potentially influencing pCR in BC patients after NACT. The results showed that serum albumin concentration, histological grading of the tumor, HER2 status, and tumor blood supply changes after two NACT cycles were significantly associated with the PCR rate in BC patients after NACT. Subsequently, the factors (*P* < 0.1) were included in the multivariate analysis. Our findings revealed that AAPR (*P* = 0.023), tumor histological grade (*P* = 0.010), HER2 status (*P* = 0.038), and changes in tumor blood supply (P = 0.032) independently predicted pCR in BC patients after NACT. (Table 3). Among them, AAPR and tumor histological grading were positively correlated with PCR; HER2 positivity and decreased tumor blood supply after two cycles of NACT were associated with a higher PCR.

**Table 3:** Univariate and Multivariate Logistic analysis for pCR of BC cancer after NACT.

Factors		Univariate analysis OR (95% CI)	*P* value	Multivariate analysis OR (95%CI)	*P* value
Age,(years)	≥ 42 vs < 42.5	1.48 (0.705–3.110)	0.300		
BMI(kg/m^2^)	≥ 24.45 vs < 24.45	1.24 (0.676–2.292)	0.483		
Menopausal status	premenopausal vs postmenopausal	0.82 (0.450–1.516)	0.536		
Therapy cycle	≥ 6 vs < 6	1.50 (0.773–2.941)	0.228		
cT	III vs I/II	0.58 (0.243–1.419)	0.237		
cN	N_X_ vs N_0_	0.72 (0.350–1.498)	0.384		
ALB	≥ 45.25 vs < 45.25	1.85 (1.003–3.429)	0.049		
ALP	≥ 73.5 vs < 73.5	1.36 (0.740–2.534)	0.317		
AAPR	≥ 0.88 vs < 0.88	1.851(0.918–3.732)	0.085	2.616(1.140–5.998)	**0.023**
NLR	≥ 2.042 vs < 2.042	1.71 (0.898–3.274)	0.102		
PLR	≥ 184.59 vs < 184.59	0.470(0.212–1.040)	0.062		
LMR	≥ 5.21 vs < 5.21	1.51 (0.790–2.891)	0.212		
Histological grade	III vs I/II	6.824(3.366–13.837)	< 0.001	3.843(1.286–10.659)	**0.010**
ER	Negative vs Positive	0.64 (0.349–1.204)	0.170		
PR	Positive vs Negative	0.63 (0.343–1.163)	0.140		
HER2	Positive vs Negative	6.406(3.265–12.570)	< 0.001	2.776(1.057–7.240)	**0.038**
Ki67	≥ 0.48 vs < 0.48	1.68 (0.882–3.225)	0.114		
Tumor diameter reduction (cm)	≥ 1.05 vs < 1.05	1.14 (0.618–2.112)	0.670		
Shape	Chang to regular vs No change	1.065(0.361–3.137)	0.910		
Adler grade	Decreased blood flow* vs No change	2.125(1.115–4.050)	0.022	2.247(1.071–4.716)	**0.032**
Boundary	Change vs No change	1.135(0.579–2.225)	0.712		
Echo	Change to high vs No change	1.262(0.447–3.564)	0.660		
Posterior acoustic	Change to shadowing vs No change	0.860(0.433–1.707)	0.666		
Calcification	Increase vs No change	0.671(0.286–1.575)	0.360		

BC: Breast cancer; NACT: Neoadjuvant chemotherapy; pCR: Pathological complete response; OR: Odds ratio; CI: Confidence interval ER:: Estrogen receptor; PR: Progesterone receptor; HER-2: Human epidermal growth factor receptor-2; ALB: Albumin; ALP: Alkaline phosphatase AAPR: albumin-to-alkaline phosphatase ratio NLR: Neutrophil-to-lymphocyte ratio; PLR: Platelet-to-lymphocyte ratio; LMR: Lymphocyte-to-monocyte ratio.* The definition of decreased blood flow is whether Adler's grading decreases after NACT.

Significant values are in bold.

### Establishment and evaluation of the nomogram model

**Figure 2:**
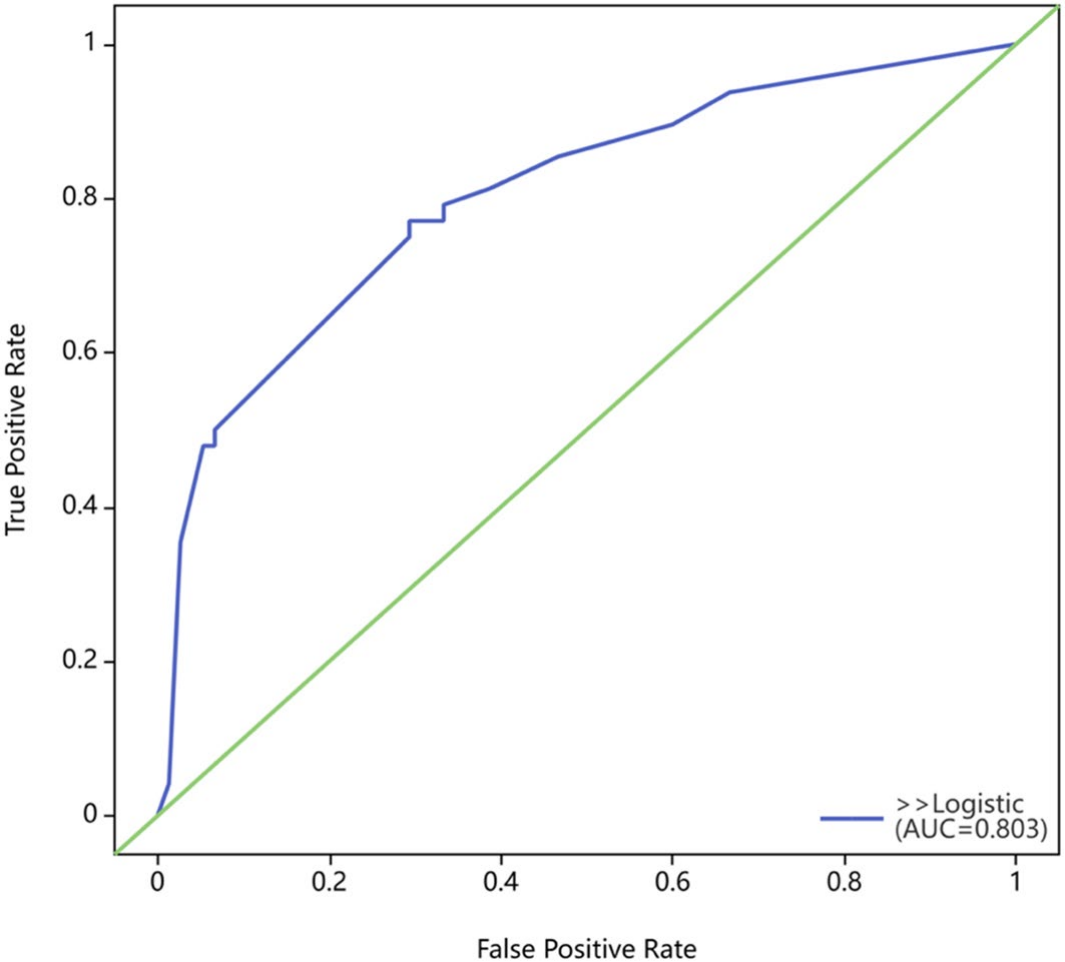
Receiver operating characteristic curve of the nomogram for predicting pCR in breast cancer patients treated with NACT.

**Figure 3:**
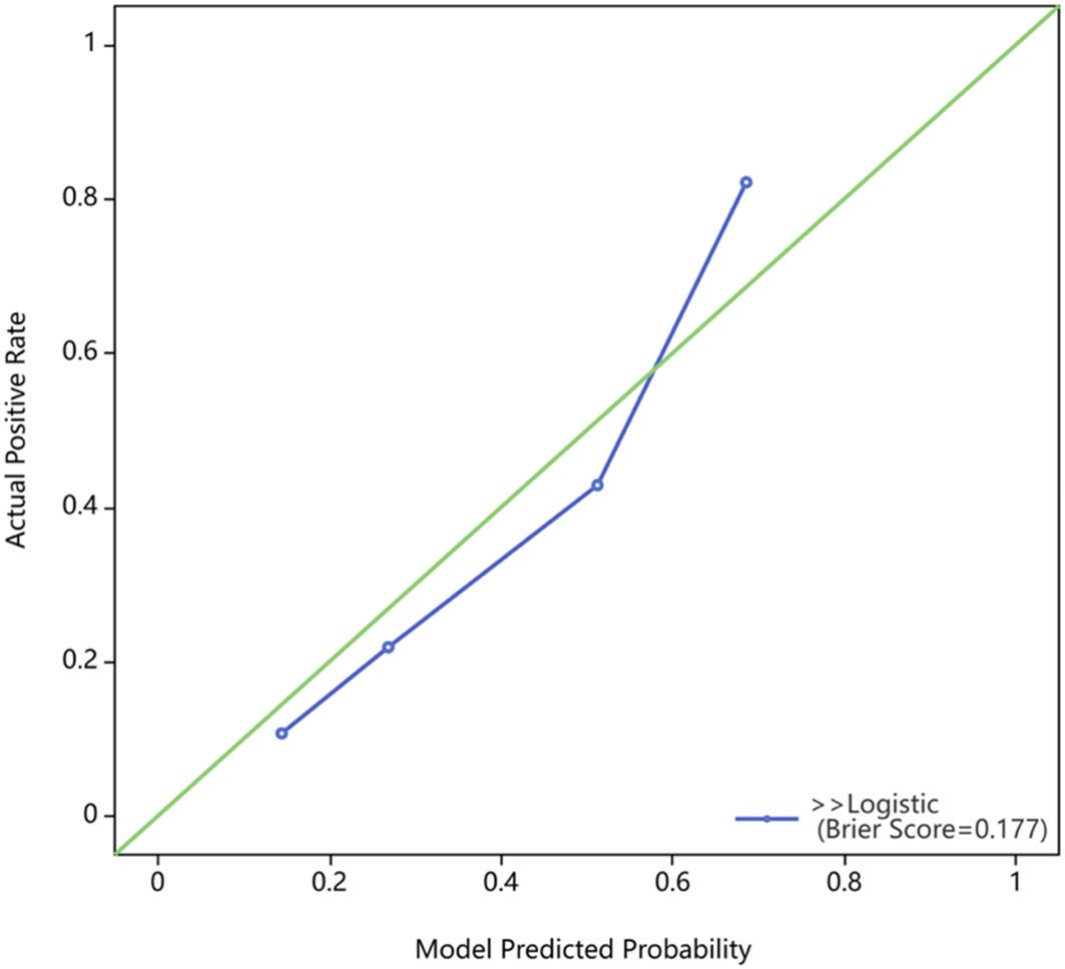
Bootstrap calibration curve of the nomogram for predicting pCR in breast cancer received NACT.

**Figure 4:**
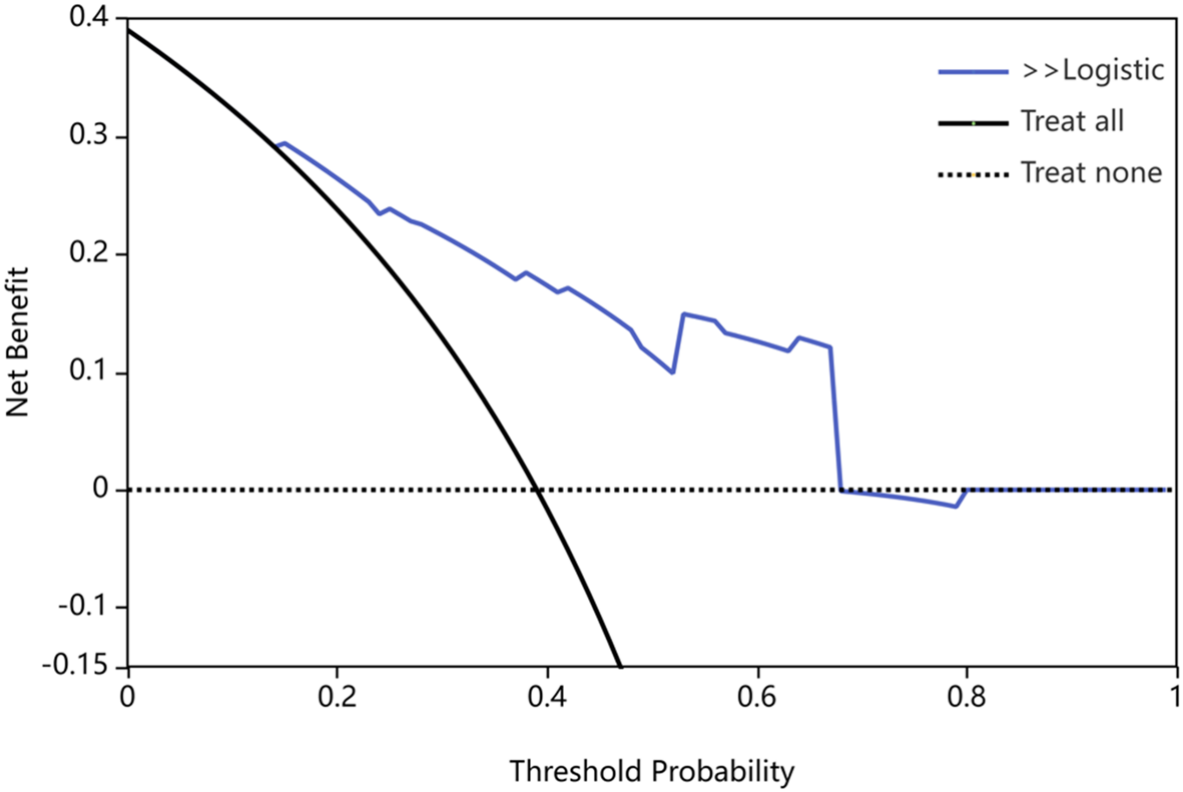
Decision curve analysis of the nomogram for predicting pCR in breast cancer after NACT.

**Figure 5:**
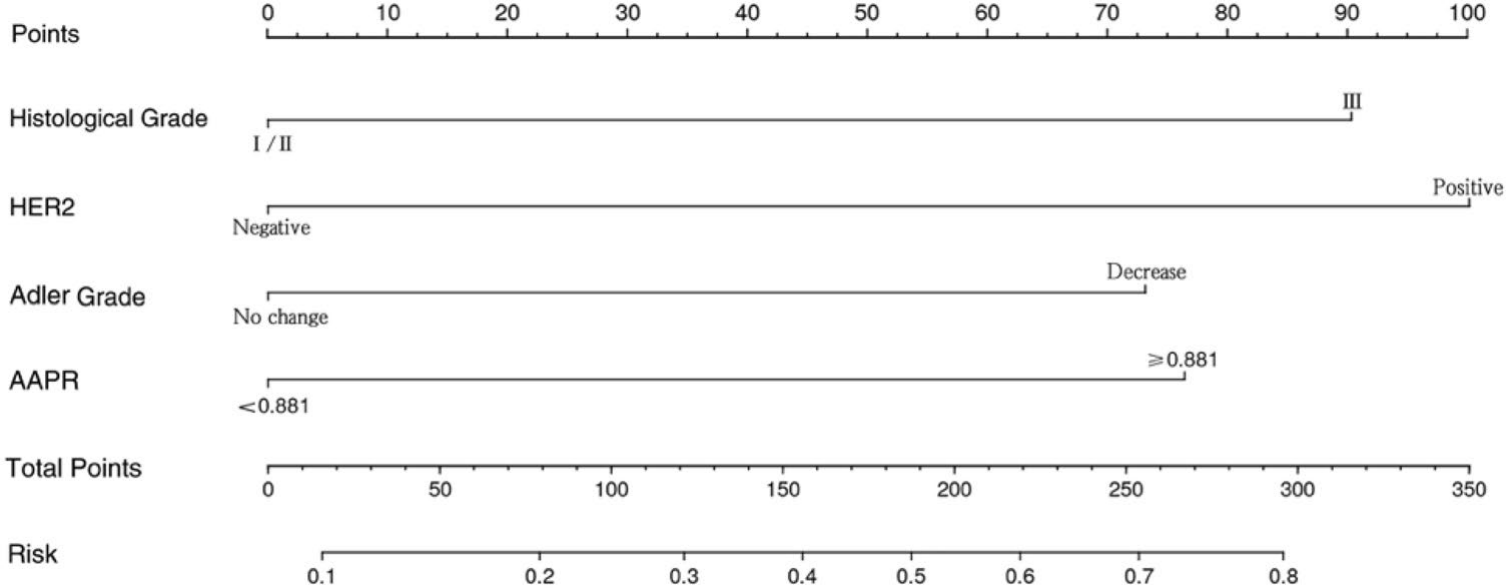
The nomogram for predicting the probability of pCR after NACT in breast cancer patients.

Using risk factors identified through multifactorial logistic regression analysis, we developed a nomogram for predicting pCR after NACT. The ROC curve was generated based on this model, yielding an area under the curve (AUC) of 0.803 (95% CI 0.723–0.884) (Fig. 2). The predictive model's concordance index (c-index) is 0.803, signifying its good discriminatory power. The calibration plot demonstrated good concordance between the predictions and actual observations (Fig. 3). The decision curve analysis (DCA) also indicated that the nomogram's pCR prediction results exhibited high accuracy for predicting pCR in NACT patients (Fig. 4). These findings imply that the nomogram is effective in predicting the pCR rate in BC patients receiving NACT (Fig. 5).

## Discussion

NACT is currently widely employed in BC treatment, with robust evidence indicating that achieving pCR after NACT is indicative of a favorable prognosis^27,28^. Our study assessed the predictive significance of the AAPR and changes in tumor blood flow for pCR in BC patients undergoing NACT. We also investigated other factors that could impact pCR. Ultimately, a nomogram prediction model based on AAPR and changes in tumor blood supply was developed using logistic regression analysis results.

Our investigation revealed that pre-chemotherapy AAPR is an independent predictor of the response to NACT in BC patients, with AAPR values demonstrating a positive correlation with pCR rates. AAPR is a novel, convenient, cost-effective, and non-invasive indicator calculated from ALB and ALP levels, providing insights into systemic inflammation and nutritional status. ALB plays a role in maintaining the equilibrium of cell proliferation and metabolism, and reduced ALB levels might be linked to compromised immune function, impacting the effectiveness of antitumor therapy^21,29^.

Prior research has demonstrated that pretreatment ALB is a prognostic factor in diverse cancers such as lung, pancreatic, gastric, and colorectal^30^. In the univariate analysis of our study, ALB exhibited a positive correlation with pCR rates (*P* = 0.049). While our study did not reveal a significant correlation between ALP and pCR, pretreatment serum ALP levels were identified as an independent prognostic factor for disease-free survival and overall survival in triple-negative breast cancer (TNBC) patients in Chen et al.'s study^31^. Initially recognized as a prognostic factor for hepatocellular carcinoma, the AAPR, calculated from ALB and ALP, has been identified as a prognostic predictor for various cancers. Nevertheless, further exploration is required to fully understand the predictive value of the AAPR in BC prognosis. Long et al. conducted a retrospective study analyzing overall survival (OS) in 746 non-metastatic BC patients, revealing that a lower albumin-to-alkaline phosphatase ratio (AAPR) was linked to a shorter OS^25^. QU et al. explored the predictive value of the AAPR for pCR in BC patients undergoing NACT. Their findings revealed that the AAPR was an independent predictor positively associated with the pCR rate. This study corroborated our findings, and the concordance index (C-index) of the prediction model was comparable in both studies: 0.803 (95% CI 0.723–0.884) in our study vs. 0.792 (95% CI 0.737–0.848) in QU et al.'s study^32^. The results of this study provide additional support for the prognostic predictive value of the AAPR in BC.

Our study also found that changes in tumor blood supply observed by ultrasound after two chemotherapy cycles were independent predictors of response to NACT treatment in BC patients. Patients with decreased tumor blood supply after two cycles of chemotherapy were more likely to achieve PCR. Information regarding the patient's tumor blood flow could be readily obtained through color Doppler ultrasound before and during NACT. Research indicates that ultrasound can dynamically monitor tumor changes during NACT and evaluate the treatment's efficacy, enabling timely adjustments to the treatment plan if necessary^33^. Changes in ultrasound parameters during treatment provide more accurate predictions of the extent of pathological remission in NACT compared to ultrasound parameters assessed before treatment in the patient's tumor. Chen et al.'s study demonstrated a significant correlation between a decrease in tumor blood supply, evaluated through ultrasound during chemotherapy, and a higher rate of pCR, consistent with our findings^26^. However, the distinction lies in the fact that Chen et al. evaluated changes in tumor blood supply before and after the entire NACT cycle (4–8 cycles), while our study specifically assessed changes after two cycles of chemotherapy. In contrast, our study anticipated the neoadjuvant efficacy of patients earlier, facilitating earlier adjustments to the treatment plan for more significant patient benefit. Stevens et al. employed enhanced MRI to explore the predictive value of changes in tumor blood flow after one NACT cycle for pathological response. Regrettably, the study did not find a predictive association between changes in breast tumor blood flow after one cycle and pathological response^34^. Moreover, enhanced magnetic resonance imaging is costly and necessitates contrast injection, while ultrasound is non-invasive, cost-effective, convenient, and rapid. Ultrasound is commonly selected for follow-up assessments of the primary tumor in patients undergoing NACT. Thus, the changes in tumor blood flow are easily accessible parameters in clinical practice. This approach avoids additional economic burdens on BC patients undergoing NACT while evaluating and predicting treatment outcomes.

Apart from AAPR and changes in tumor blood supply after two cycles, multifactorial analysis revealed HER2 status and tumor histological grading as independent predictors. In line with earlier observations, patients with positive HER2 status and high tumor histological grading exhibited elevated rates of pCR^4,35^. Utilizing the readily available clinical indicators mentioned above, we constructed a nomogram to predict pCR in BC patients undergoing NACT. To our knowledge, this is the first model that combines AAPR with changes in tumor blood supply to predict pCR in BC patients receiving NACT.

The nomogram is a simple and effective tool for predicting prognosis. The C-index of the nomogram was 0.803 (95% CI 0.723–0.884), and the AUC value of the ROC curve was 0.803, which indicated that the nomogram prediction model had good accuracy in the prediction of pCR. In addition, the calibration plot and DCA curve also indicated that the prediction model had good predictive ability. Therefore, this study provides a novel, simple, and feasible prediction model for BC patients with pCR after NACT.

However, there are limitations in this study. Firstly, BC is a heterogeneous disease, and the relatively small sample size hinders the execution of subgroup analyses, particularly for independent investigations of hormone receptor-positive BC with low pCR rates. Secondly, the study population was drawn from a single center and solely validated through internal validation. Additional external validation from diverse centers is essential to assess the model's accuracy. Finally, this study was retrospective, posing challenges in obtaining more comprehensive clinical information. The analysis focused only on common clinicopathological factors, laboratory indicators, and ultrasound parameters. Thus, enlarging the sample size and incorporating more indicators are imperative for enhancing the prediction model in subsequent research.

## Conclusion

This study developed a simple, innovative, and easily applicable nomogram prediction model utilizing four independent predictors: AAPR, changes in tumor blood flow, HER2 status, and tumor histological grading. The model demonstrates effective predictive capabilities for pCR after NACT, integrating changes in ultrasound image parameters with clinicopathological indicators. Further exploration of its potential is warranted in future research.

### Supplementary Information

Supplementary Information.

## Data Availability

Any request for data and materials should be made in writing to the corresponding author, and these will be considered.
