# Supplementary material for: Predicting pathological complete response after neoadjuvant chemotherapy in breast cancer by clinicopathological indicators and ultrasound parameters using a nomogram
Source: Sci Rep. 2024 Jul 16;14:16348. doi: 10.1038/s41598-024-64766-2 (PMC11252377; doi:10.1038/s41598-024-64766-2)
Supplement: Supplementary file 1 — Supplementary Information. [file 41598_2024_64766_MOESM1_ESM.docx]

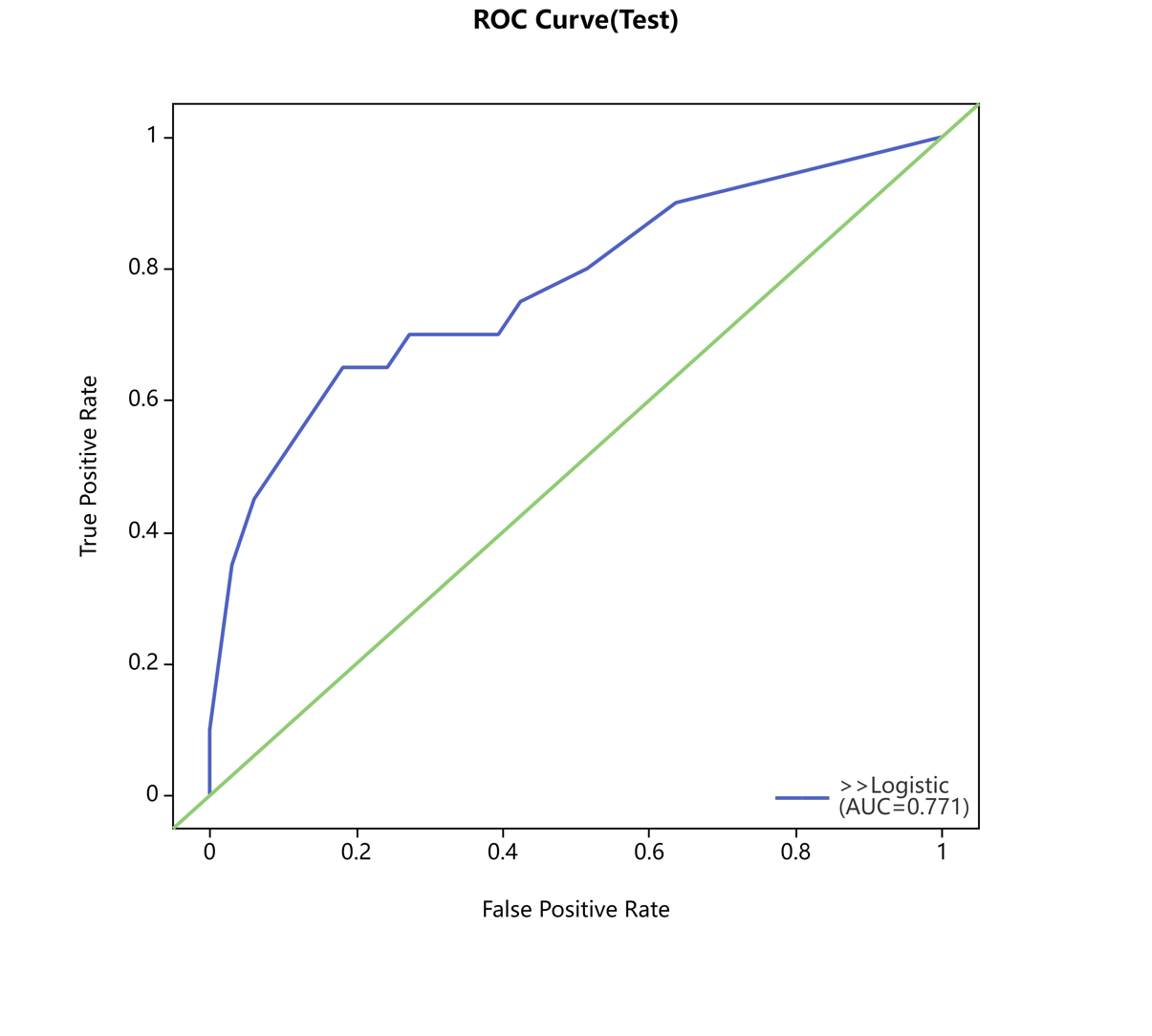


Figure 1

Receiver operating characteristic curve(test) of the nomogram for predicting pCR in breast cancer patients treated with NACT.


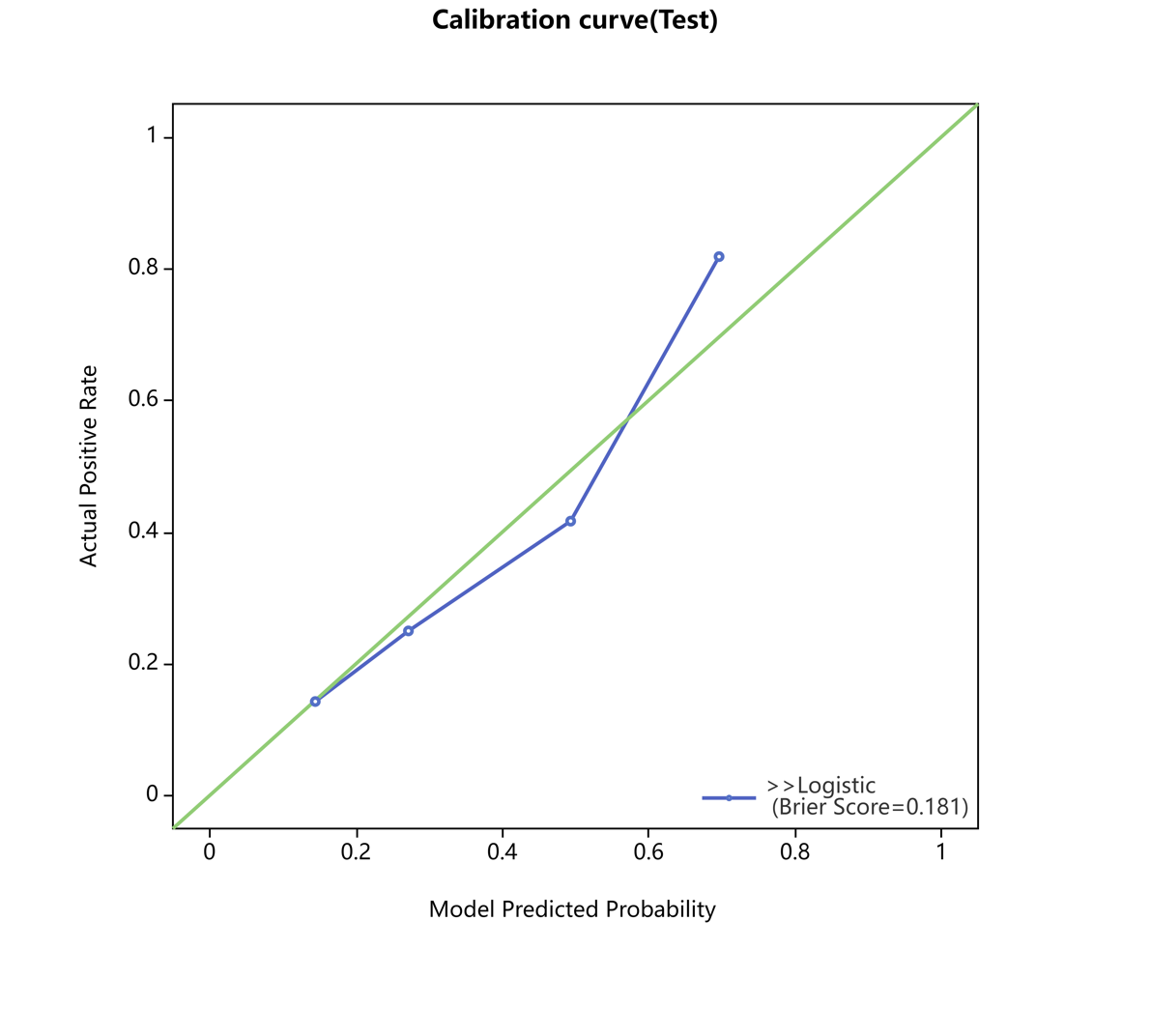


Figure 2

Bootstrap calibration curve(test) of the nomogram for predicting pCR in breast cancer received NACT.


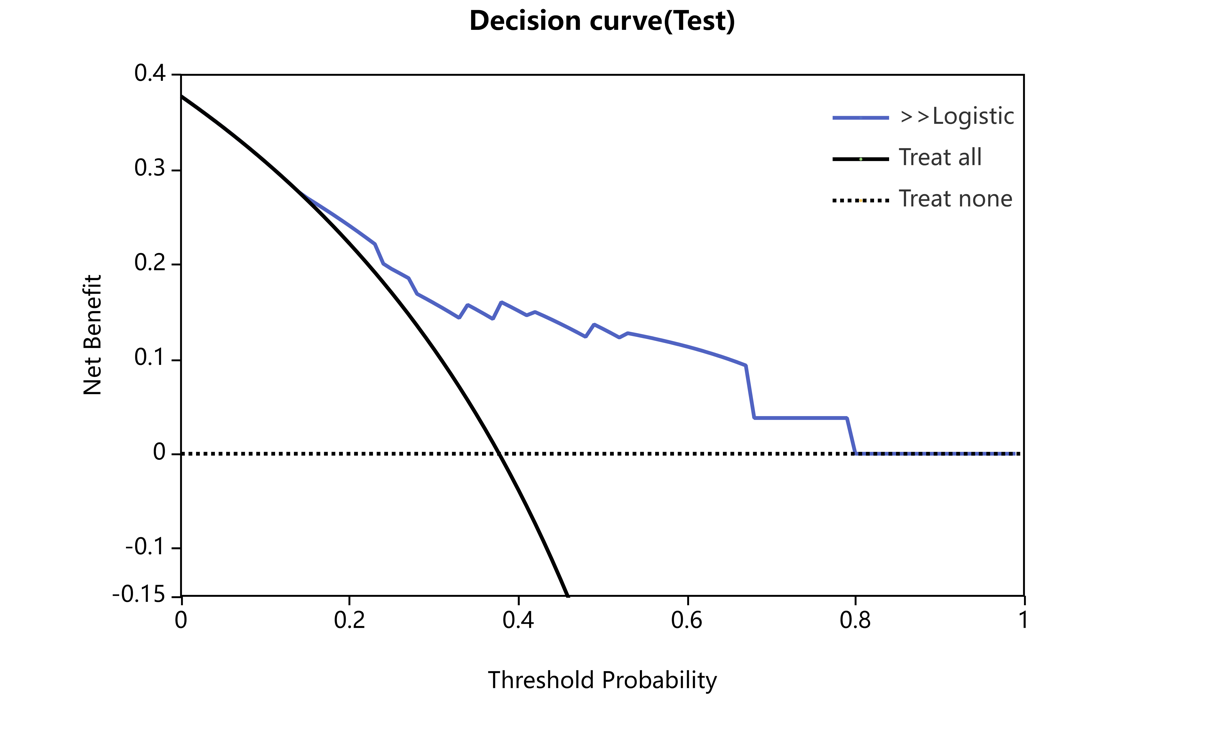


Figure 3

Decision curve(test) analysis of the nomogram for predicting pCR in breast cancer after NACT.
